# Wide and narrow QRS complexes during flutter. What’s the mechanism?

**DOI:** 10.1016/j.hrcr.2021.03.003

**Published:** 2021-03-09

**Authors:** Gabriela Răileanu, László Sághy, Péter Kupó, Róbert Pap

**Affiliations:** Second Department of Internal Medicine and Cardiology Center, Medical School, University of Szeged, Szeged, Hungary

**Keywords:** Atrial flutter, Left bundle branch block morphology, Premature atrial beat, Recurrent palpitations, Wide QRS tachycardia

## Introduction

Intermittent wide QRS complexes with left bundle branch block (LBBB) morphology during atrial flutter usually represent aberration. However, an open mind should be kept with regard to less common mechanisms as presented in this unusual case.

## Case report

**Figure 1:**
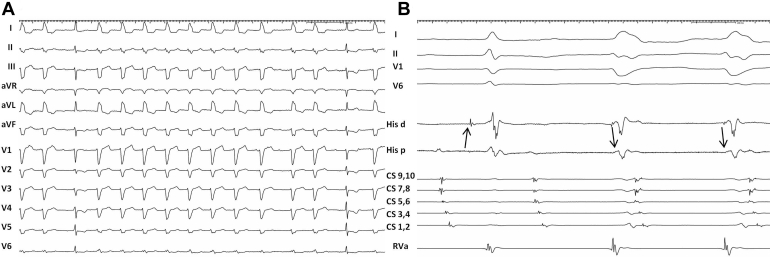
**A:** Baseline electrocardiogram showing atrial flutter with wide, left bundle branch block morphology QRS complexes interspersed with narrow QRS complexes. Sweep speed 25 mm/s. **B:** During spontaneous initiation of wide QRS complex tachycardia, His signal “moves” after the beginning of the QRS complex, and distal His signal comes earlier than proximal His signal. Sweep speed 125 mm/s. CS = coronary sinus; His = His bundle; RVa = right ventricular apex.

A 72-year-old woman, with a history of pulmonary vein isolation and cavotricuspid isthmus ablation for atrial fibrillation and flutter (AFL), presented to our department for recurrence of palpitations. Her history was also remarkable for a previous episode of wide QRS tachycardia with a rate of 110 beats/min, terminated by carotid sinus massage. The resting electrocardiogram showed AFL with wide QRS complexes, interspersed by normal QRS complexes (Figure 1A).

**Figure 2:**
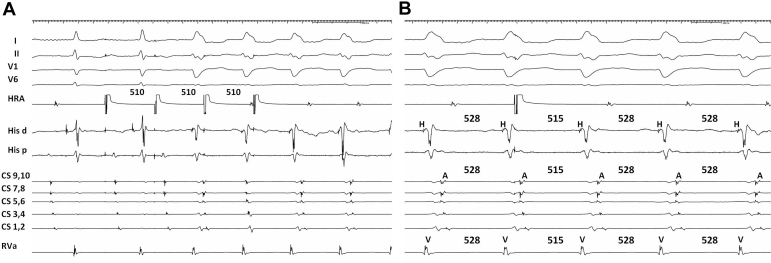
**A:** Wide QRS complex tachycardia induced by high right atrial burst pacing. Sweep speed 65 mm/s. **B:** Atrial premature stimulation delivered from the high right atrium during wide complex tachycardia. Shown are His-to-His, septal atrium-to-atrium, and right ventricle-to-ventricle intervals. Sweep speed 100 mm/s. CS = coronary sinus; His = His bundle; HRA = high right atrium; RVa = right ventricular apex.

Mapping revealed a left atrial flutter, which terminated during ablation at the left atrial anterior wall. After termination of the AFL, a tachycardia with identical wide QRS complexes spontaneously initiated (Figure 1B) and was also induced by burst pacing the atrium (Figure 2A). This tachycardia was reset by a late atrial extrastimulus (Figure 2B). What is the mechanism of wide QRS complexes during AFL and the wide complex tachycardia?

## Discussion

Wide QRS complexes during flutter show LBBB morphology; their differential includes intermittent aberration and pre-excitation. During initiation of wide complex tachycardia with identical QRS morphology, the His deflection is seen to “move” inside the QRS complex (Figure 1B). At the same time proximal-to-distal His activation changes to distal-to-proximal. These observations rule out aberration and leave ventricular tachycardia or pre-excited tachycardia as options for the mechanism of the wide complex tachycardia.

An atrial extrastimulus from the high right atrium, delivered when atrial tissue around the atrioventricular (AV) node is refractory, pre-excites the next ventricular activation, with identical QRS morphology, and resets the tachycardia with unchanged VA interval and atrial activation sequence (Figure 2B). This is diagnostic of antidromic AV reentrant tachycardia utilizing an accessory pathway (AP) from the right atrial free wall.^1^^,^^2^ An atrial origin was further excluded by a VAV response after ventricular overdrive pacing.

The unusual observation in this case is that the QRS complex is either fully pre-excited or normal during both atrial flutter and pacing. The lack of fusion complexes can be explained by the insertion of the AP directly into the proximal right bundle, as evidenced by an early retrograde His signal during antidromic AV reentrant tachycardia (Figure 1B). Thereby early retrograde invasion of the other limb occurs by impulses traveling down either the AV node or the pathway. This, together with the long conduction time of the AP (reflected in the long cycle length of the tachycardia), prevents pre-excitation during slow atrial rates. During faster pacing and atrial flutter the slightly shorter Wenckebach cycle length of the AP and retrograde penetration of the normal conduction system produce fully pre-excited QRS complexes, until 2 consecutive atrial impulses block in both limbs, providing time for the normal conduction system to recover and conduct the following impulse with a normal QRS (Figure 1A).

**Figure 3:**
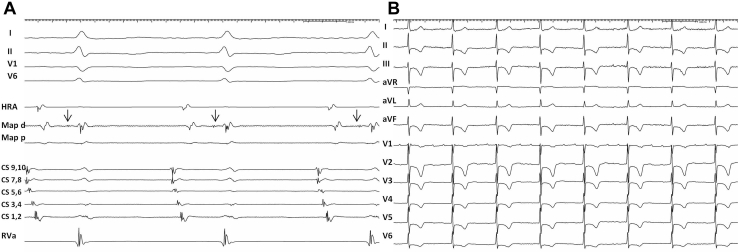
**A:** “M potential” recorded while mapping the lateral aspect of the tricuspid annulus. Unusual atrial activation sequence during sinus rhythm likely due to advanced atrial scarring. Sweep speed 150 mm/s. **B:** Second flutter induced after Mahaim pathway ablation conducted exclusively with normal QRS complexes. Sweep speed 25 mm/s. CS = coronary sinus; HRA = high right atrium; Map = lateral tricuspid annulus; RVa = Right ventricular apex.

The slowly conducting, atriofascicular Mahaim AP was mapped to the lateral tricuspid annulus, guided by a His-like Mahaim potential (Figure 3A). Ablation at this site eliminated the wide QRS tachycardia and when a second flutter was induced, this conducted exclusively with normal QRS complexes (Figure 3B).

This case demonstrates that Mahaim fibers can masquerade as rate-dependent LBBB aberration during atrial tachycardia.

**Key Teaching Points**

• Wide QRS complexes with left bundle branch block morphology during atrial flutter can be suggestive either for intermittent aberration or for pre-excitation.
• His activation sequence and its relationship with the beginning of the QRS complex during wide QRS tachycardia are important clues to rule out aberration.
• An atrial extrastimulus delivered in the His refractory period during wide QRS complex tachycardia, which advances the next ventricular activation and resets the tachycardia, is suggestive for the presence of an accessory pathway.
